# Supplementary material for: Cryo-EM structures of Na+-pumping NADH-ubiquinone oxidoreductase from Vibrio cholerae
Source: Nat Commun. 2022 Jul 26;13:4082. doi: 10.1038/s41467-022-31718-1 (PMC9325719; doi:10.1038/s41467-022-31718-1)
Supplement: Supplementary file 1 — Supplementary Information [file 41467_2022_31718_MOESM1_ESM.pdf]

Supplementary Information

Cryo-EM structures of Na^+-pumping NADH-ubiquinone oxidoreductase from *Vibrio cholerae*

Jun-ichi Kishikawa, Moe Ishikawa, Takahiro Masuya, Masatoshi Murai, Yuki Kitazumi, Nicole L. Butler, Takayuki Kato, Blanca Barquera, and Hideto Miyoshi

*To whom correspondence should be addressed: Hideto Miyoshi, Division of Applied Life Sciences, Graduate School of Agriculture, Kyoto University, Kyoto 606-8502, Japan,

Tel: (+81)-75-753-6119, E-mail: miyoshi.hideto.8e@kyoto-u.ac.jp

Supplementary Fig. 1
The binding sites of inhibitors and the UQ head-ring identified by photoaffinity labeling

Supplementary Fig. 2
Flow charts for image processing of three Na^+-NQR preparations

Supplementary Fig. 3
The local resolution of the obtained Na^+-NQR maps

Supplementary Fig. 4
Biochemical characterization of the purified Na^+-NQR

Supplementary Fig. 5
Electrostatic interactions between NqrF and NqrA in the 3 states

Supplementary Fig. 6
Structure of the contact area between NqrA and NqrB

Supplementary Fig. 7
Comparison of the Na^+-NQR structures between aurachin D-42-bound and non-bound states

Supplementary Fig. 8

The binding manner of aurachin D-42 and korormicin A in NqrB

Supplementary Fig. 9

N-Acyl-*N*-alkyl sulfonamide chemistry

Supplementary Fig. 10

The binding site of the UQ head-ring in the cryo-EM structure

Supplementary Fig.11

Locations of NqrB-Asp397, NqrD-Asp133, and NqrE-Glu95 in the cryo-EM structure of Na^+-NQR

Supplementary Fig. 12

Structure-inhibition relationship of korormicin A derivatives

Supplementary Table 1

Statistics for Cryo-EM data, refinement, and validation of Na^+-NQR.

Supplementary Table 2

Statistics for Cryo-EM data, refinement, and validation of Na^+-NQR^{AD42} and Na^+-NQR^{KA}.

Supplementary Table 3

Comparison of the r.m.s.d values for each subunit between the cryo-EM and crystallographic structures

Supplementary Discussion (Supplementary Figs. 13 to 18)

Equilibrium binding model of ^{125}I-incorporated inhibitor and competitor

Uncropped gel scans of the purified Na^+-NQR (for Supplementary Fig.4)

(A)

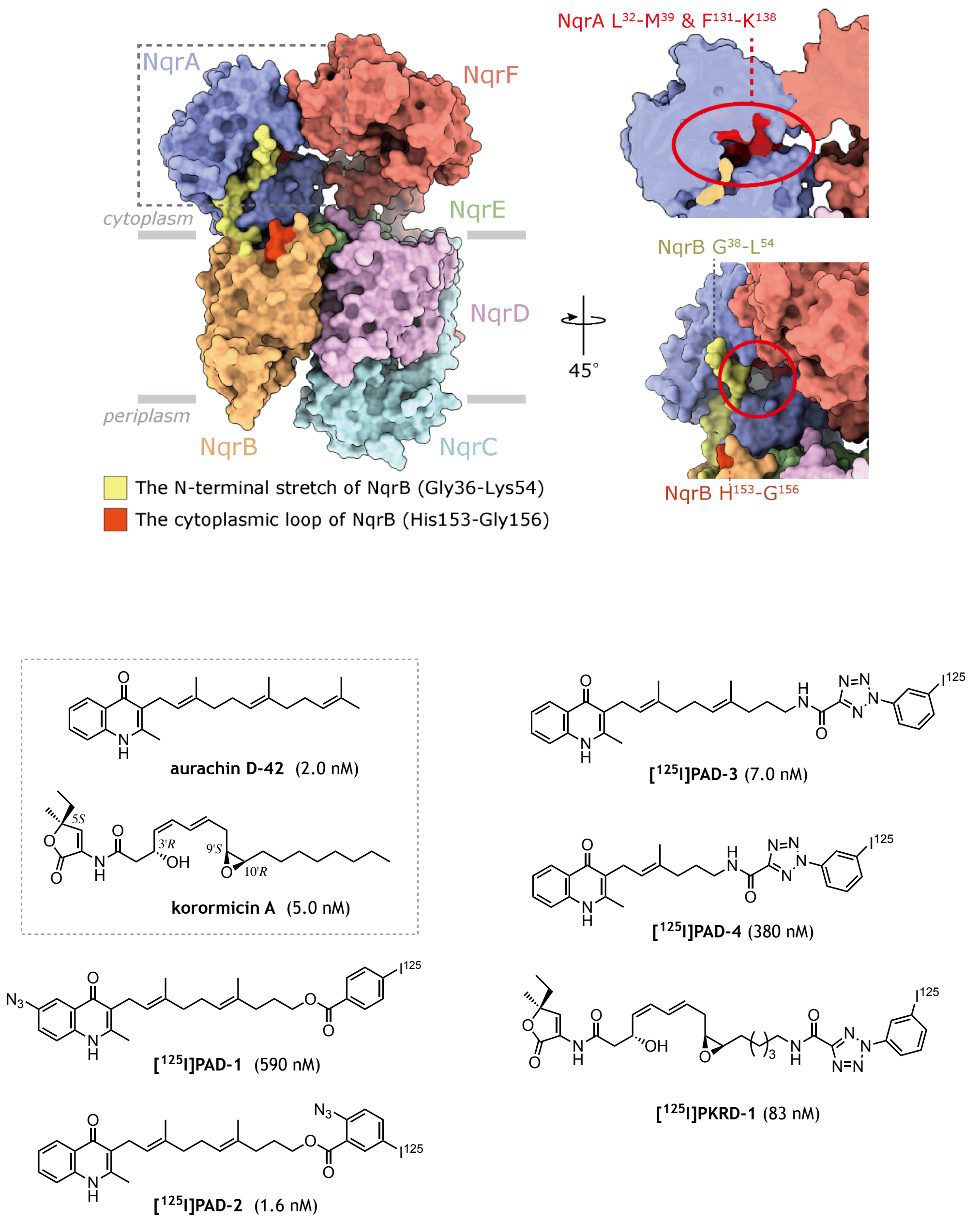

Supplementary Fig. 1: **The binding sites of inhibitors and the UQ head-ring identified by photoaffinity labeling.** (**A**) The binding sites of inhibitors in NqrB and the UQ head-ring in NqrA are shown in the X-ray crystallographic structure (*16*, PDB ID: 4P6V). The korormicin A derivatives ([^{125}I]PKRD-1) and aurachin D derivatives ([^{125}I]PAD-1−[^{125}I]PAD-4) bind to the region (Try23−Lys54 in *yellow*) in the protruding N-terminal stretch starting with TMH 1 of NqrB and/or a part of the cytoplasmic loop (His153−Gly156 in *red*) connecting TMHs 2–3 of NqrB. The UQ head-ring binds to the cytoplasmic region of NqrA (Leu32−Met39 and Phe131−Lys138, indicated by a red circle). (**B**) The structures of the photoreactive inhibitors, which were used in the previous studies (*18* and *19*), are shown. The average IC_{50} value of each inhibitor, which were determined with 1.0 nM Na^+-NQR, are shown in the parentheses.

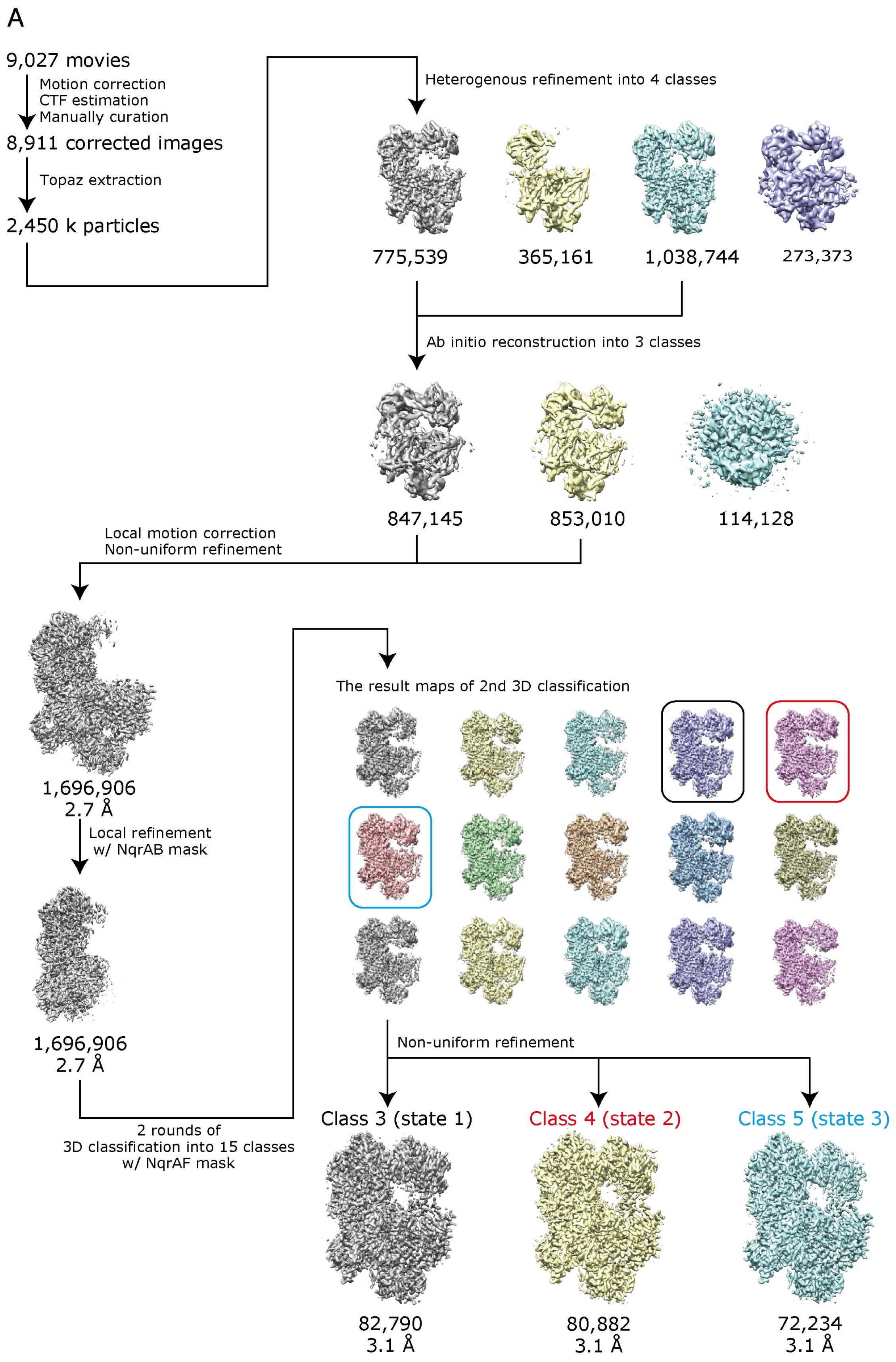

Supplementary Fig. 2 (continued)

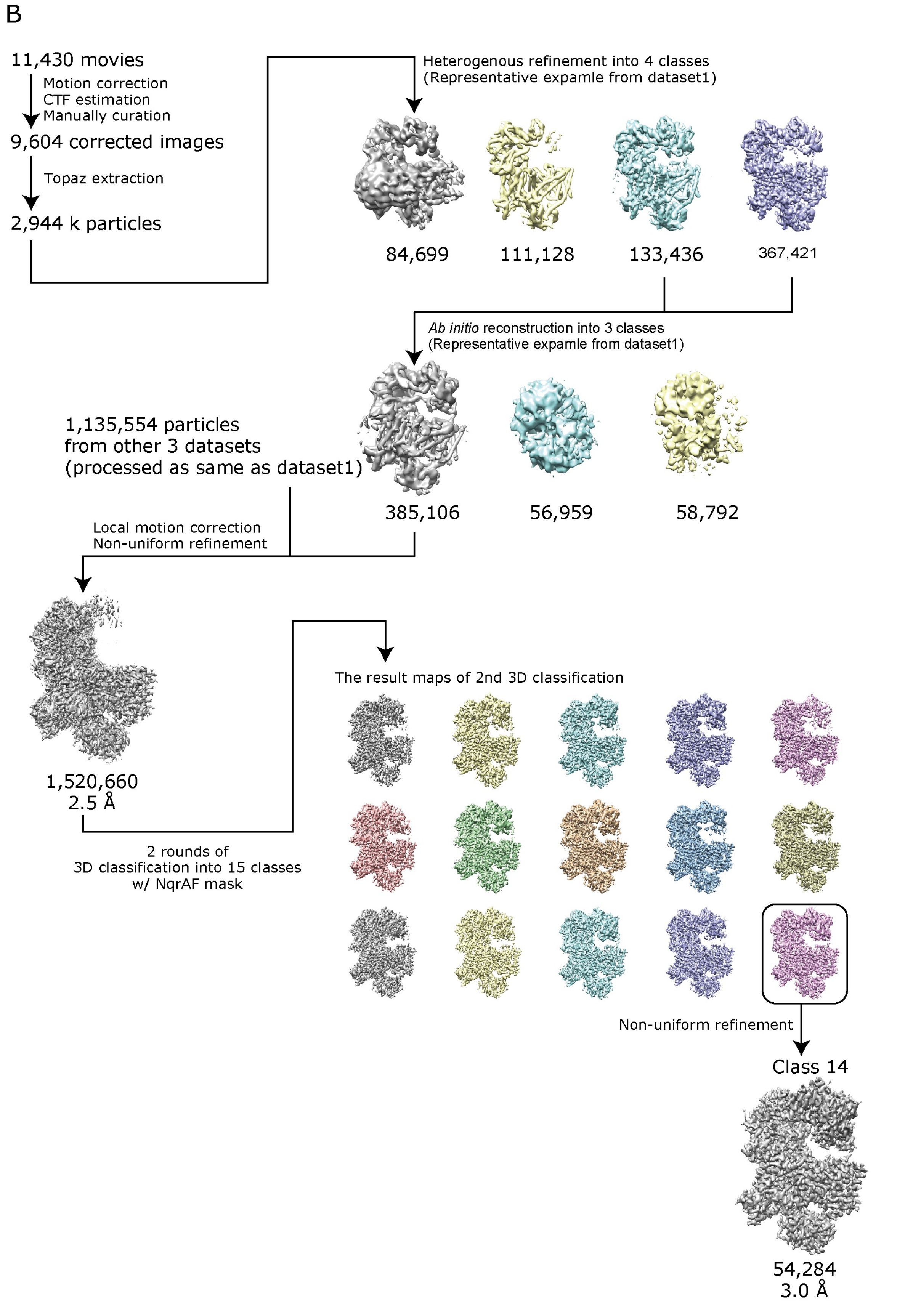

Supplementary Fig. 2 (continued)

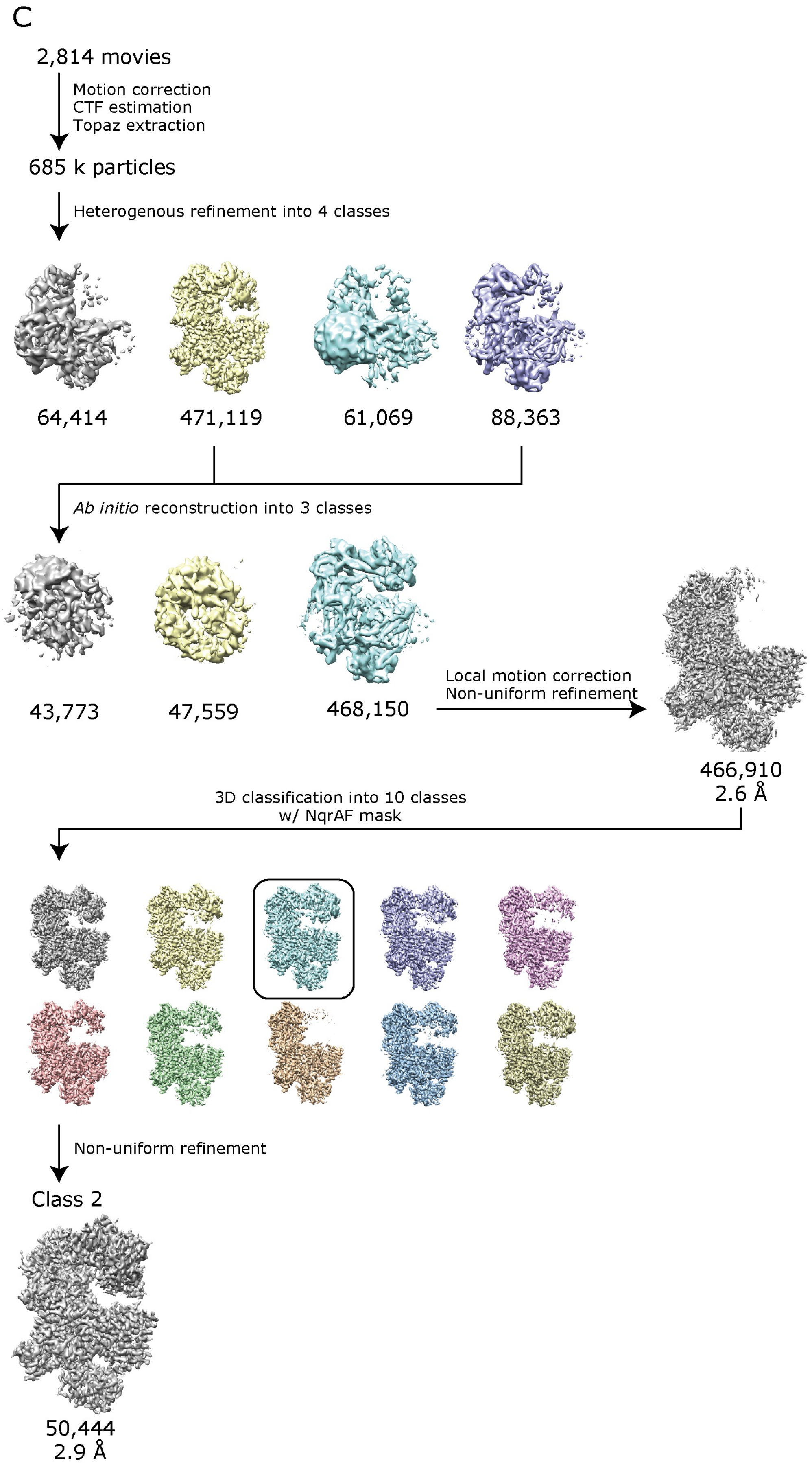

Supplementary Fig. 2 (continued)

Supplementary Fig. 2: Flow charts for image processing of three Na^+-NQR preparations. (**A**) Na^+-NQR without inhibitor, (**B**) Na^+-NQR with bound aurachin D-42 (Na^+-NQR^{AD42}), and (**C**) Na^+-NQR with bound korormicin A (Na^+-NQR^{KA}). For the dataset of Na^+-NQR^{AD42} (total 11,430 movies), the dataset was separated into four set. The image processing of each set was conducted according to the flow chart. Representative results of the four dataset is shown in a panel **C**. The selected particles from each dataset were merged before Homogenous refinement.

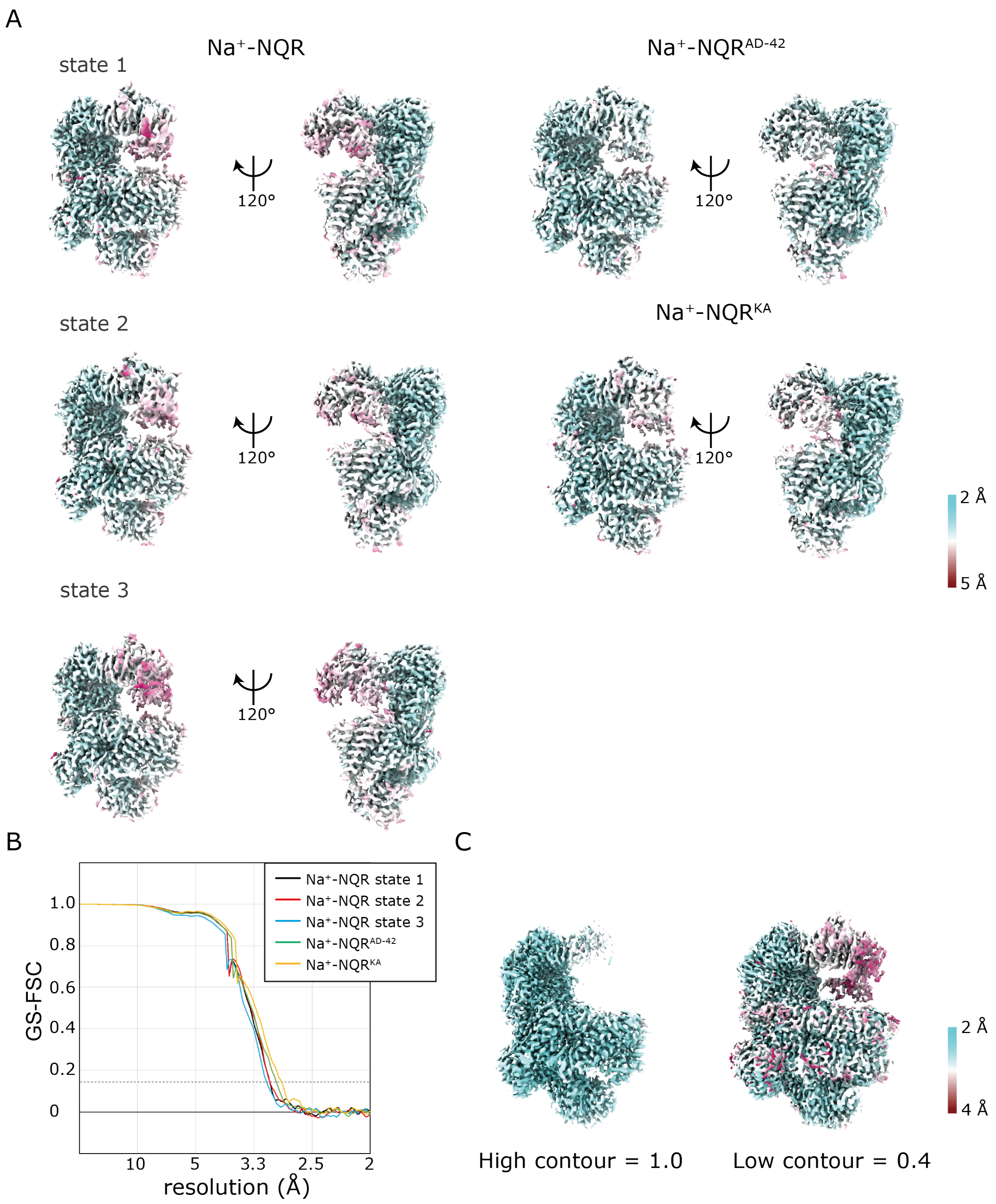

Supplementary Fig. 3: The local resolution of the obtained Na$^+$-NQR maps.

(**A**) Cryo-EM density maps of Na$^+$-NQR (states 1–3), Na$^+$-NQR^{AD42}, and Na$^+$-NQR^{KA}. The maps are colored according to local resolution as indicated in the color bar. (**B**) Gold-standard Fourier shell correlation (GS-FSC) curves for each Na$^+$-NQR, using GS-FSC = 0.142 for resolution criterion (dotted line). (**C**) The consensus map for Na$^+$-NQR is represented as solid surfaces. The map was obtained Non-Uniform refinement using all selected particles (**Supplementary Fig. 1**). The maps are colored by local resolution indicated as color key. The contour levels are set to high (*left*) and low (*right*). The density corresponding to the hydrophilic domain of NqrF are weak and has low resolution compared to other subunits.

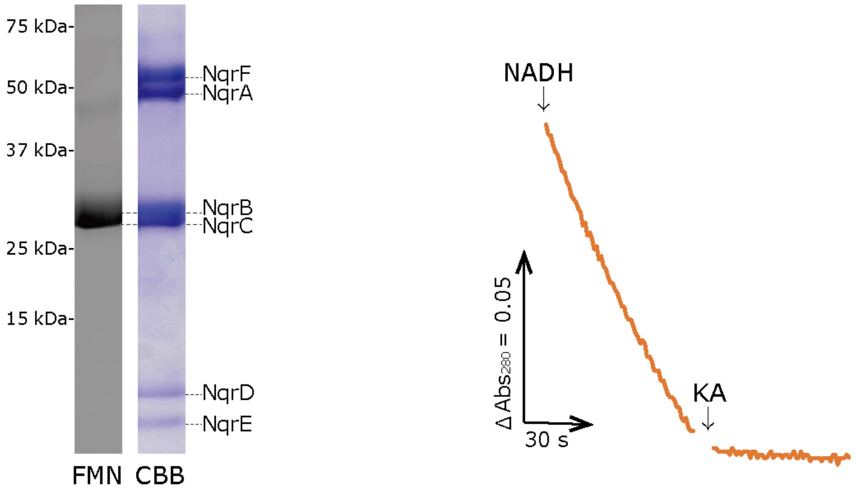

Supplementary Fig. 4: Biochemical characterization of the purified Na^+-NQR.
A left panel: SDS-PAGE of the purified *V. cholerae* Na^+-NQR. The subunits were stained with CBB R-250. FMNs in the NqrB and NqrC subunits were visualized using bio-imaging analyzer Typhoon FLA-9500 (Cytiva) using a 473 nm light source and an LPB emission filter (emission wavelengths shorter than 510 nm are cut off). Gel images are representative of three different enzyme preparations. A right panel: The NADH-UQ_1 oxidoreductase activity of Na^+-NQR (1.0 nM) was almost completely inhibited by korormicin A (KA, 1.0 μM). Trace is representative of two separate assays for each of three different enzyme preparations.

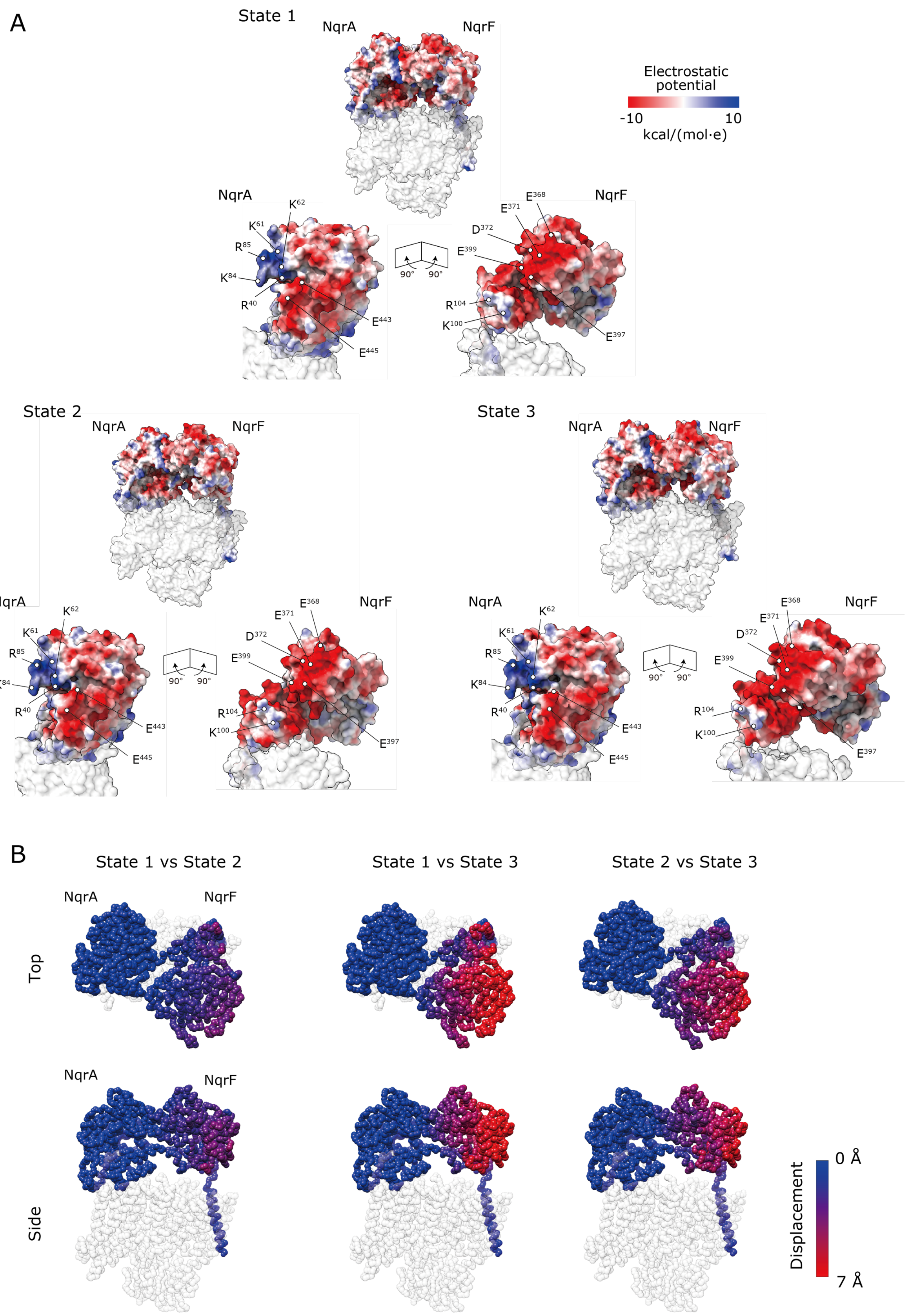

Supplementary Fig. 5 (continued)

Supplementary Fig. 5: Electrostatic interactions between NqrF and NqrA in the 3 states. (**A**) The structures of the interacting area between NqrF and NqrA in states 1–3. The structures of the interacting area are almost identical one another irrespective of significant structural differences of the rest of the hydrophilic parts of NqrF. The positive patch formed by Arg40, Lys61, Lys62, Lys84, and Arg85 of NqrA interacts with the negative patch formed by Glu368, Glu371, Asp372, Glu397, and Glu399 of NqrF in the three states. Negative tips of NqrA (Glu443 and Glu445) are in close proximity to positive tips of NqrF (Lys100 and Arg104). (**B**) Each state of Na^+-NQR was superimposed on the NqrB subunit. NqrA and NqrF are represented as a sphere model and colored by displacement values between the three states calculated for the Cα atoms; *blue* (small changes) to *red* (large changes). Other subunits are shown in semi-transparent.

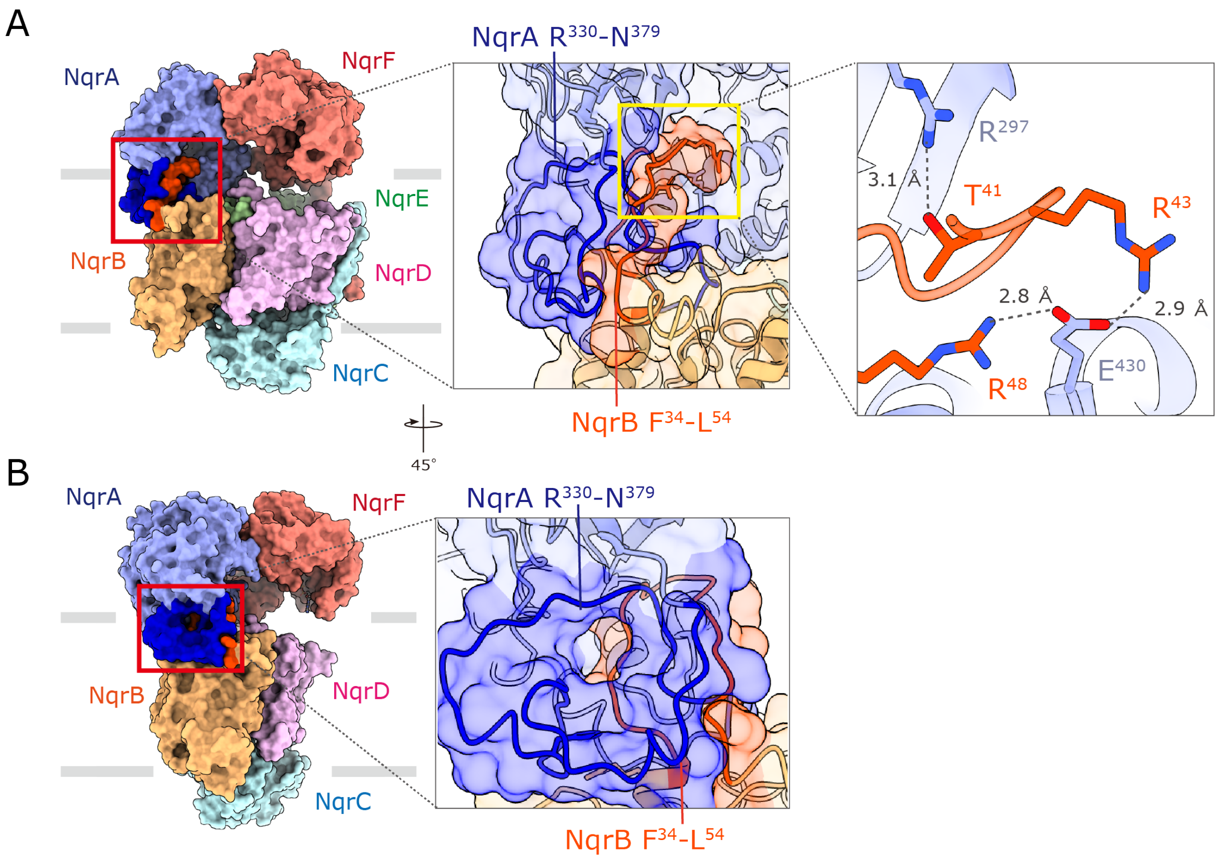

Supplementary Fig. 6: Structure of the contact area between NqrA and NqrB.
(**A**) The overall and enlarged views of the contact area between the C-terminal region of NqrA (Arg330−Asn379, in *dark blue*) and the protruding part of the N-terminal region of NqrB (Phe34−Leu54, in *red*). (**B**) The view of the contact area from a different angle.

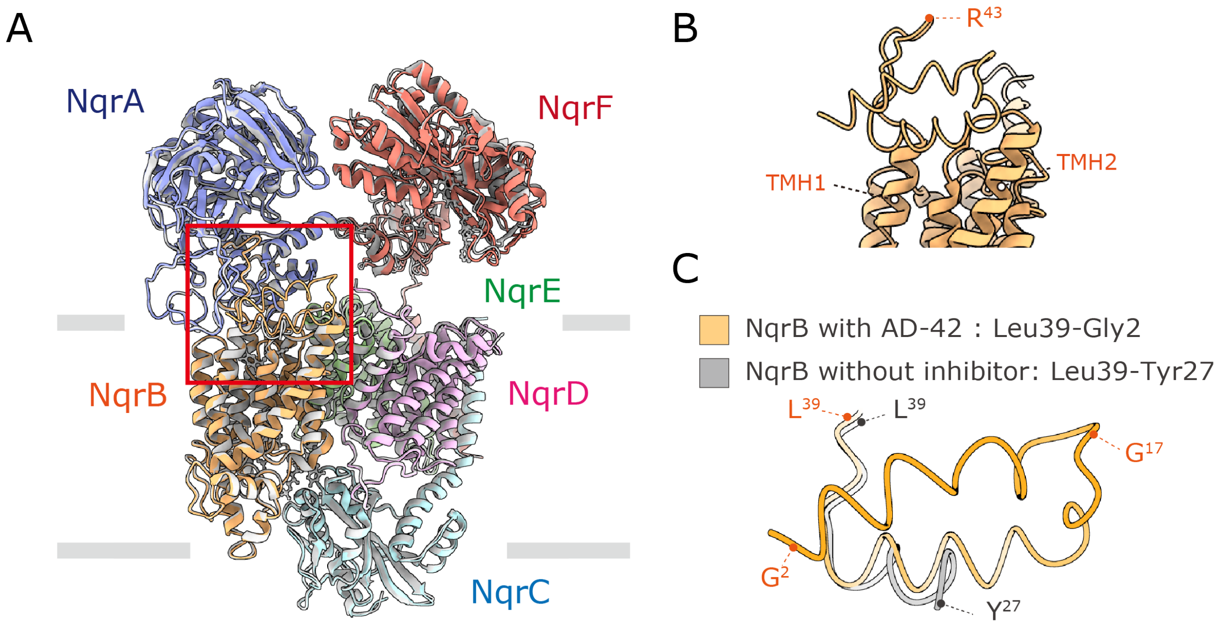

Supplementary Fig. 7: Comparison of the Na$^+$-NQR structures between aurachin D-42-bound and non-bound states.

(**A**) Overlay of Na$^+$-NQR structure with bound aurachin D-42 (*color*) with the structure in the absence of inhibitor (*gray*). The two structures are almost identical except for the N-terminal region of NqrB indicated by a red square. (**B**) The structure of the protruding N-terminal stretch starting with TMH 1 of NqrB in Na$^+$-NQR with bound aurachin D-42. The N-terminal stretch first protrudes from the membrane phase and then turns back toward the membrane by bending at NqrB-Arg43. (**C**) Close-up view of the N-terminal region of NqrB. The region Gly2–Leu26 is disordered in the absence of the inhibitor (*gray*).

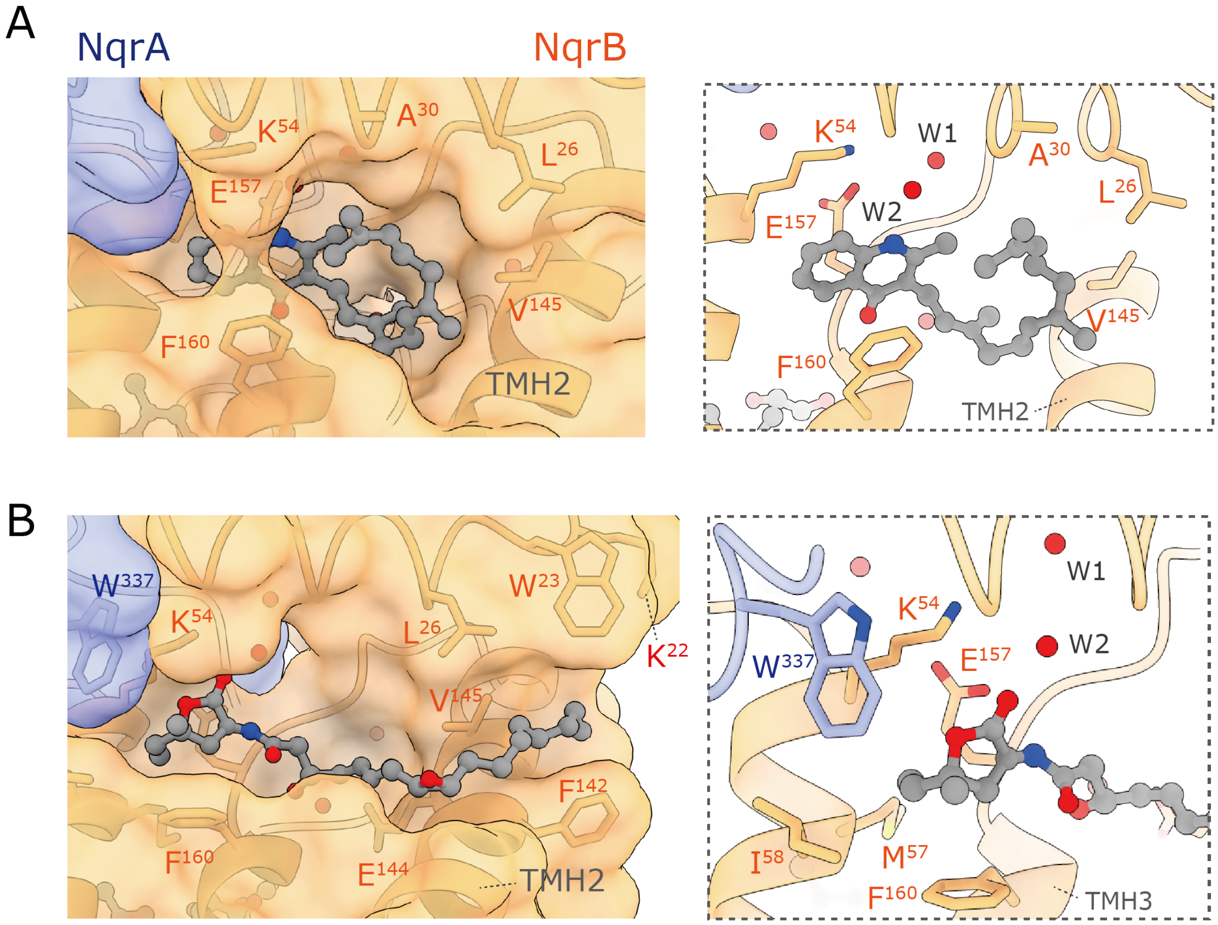

Supplementary Fig. 8: The binding manner of aurachin D-42 and korormicin A in NqrB. (**A**) Close-up view of the binding site of aurachin D-42. NqrB-Phe160 may be involved in a π-stacking interaction with the quinolone ring, which locks aurachin D-42 inside the binding cavity. (**B**) Close-up view of the binding site of korormicin A. The steric obstruction between the alkyl branches (5-CH_3/C_2H_5) on the lactone ring and the cavity wall formed by NqrB-Met57 and -Ile58 on TMH 1, NqrB-Phe160 on the TMH 3, and NqrA-Trp337 may fix the conformation of the lactone ring. The alkyl side chain extends toward a hydrophobic cleft composed of Trp23, Leu26, Phe142, and Val145 of NqrB. Water molecules are shown as *red spheres*.

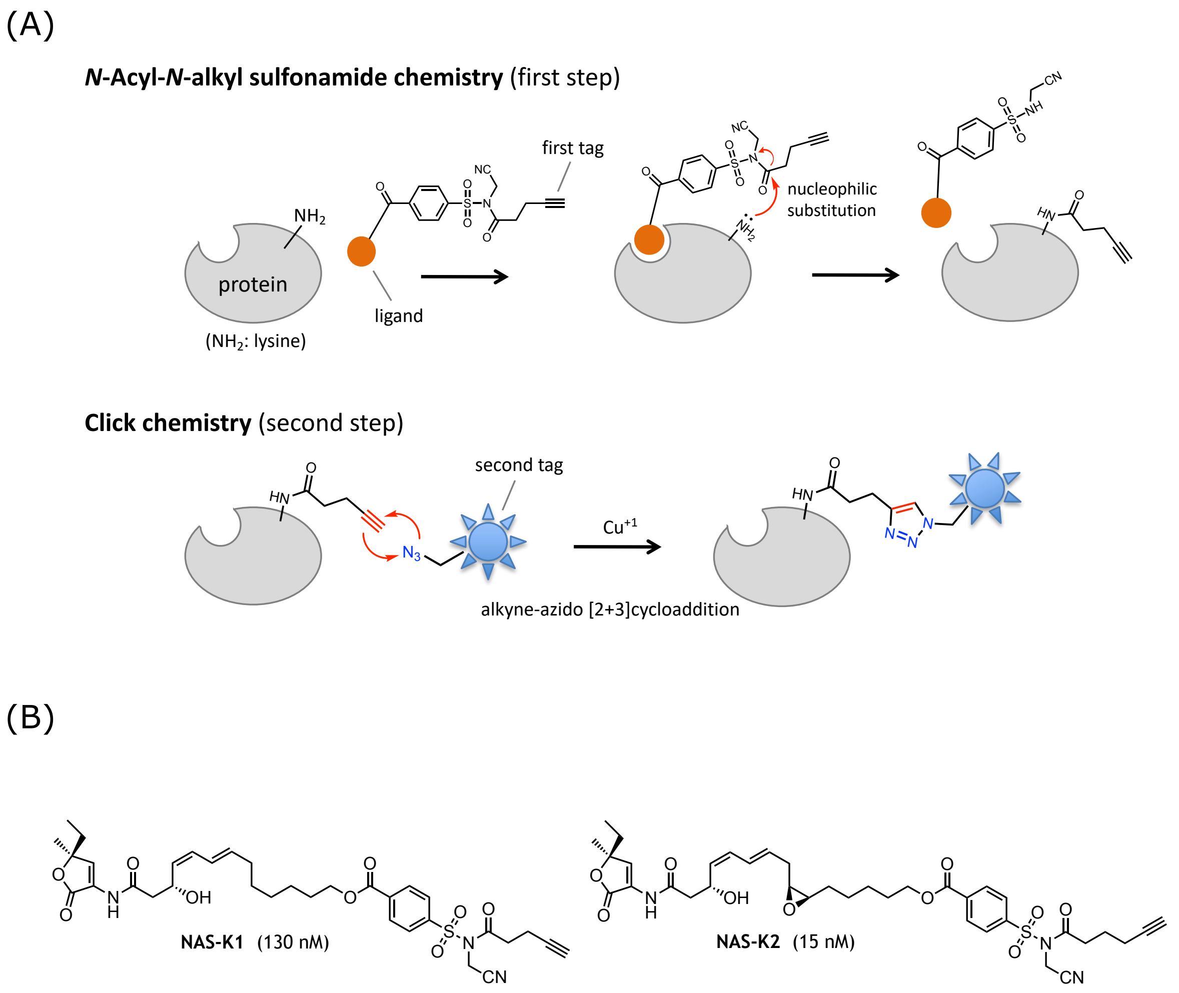

Supplementary Fig. 9: *N*-Acyl-*N*-alkyl sulfonamide chemistry. (**A**) *N*-Acyl-*N*-alkyl sulfonamide chemistry is schematically shown. A first tag attached to the sulfonamide moiety is introduced to lysine via *N*-acyl-*N*-alkyl sulfonamide chemistry (*27*). Then, the reacted lysine can be identified by proteomic analyses after a second tag (e.g. fluorescent tag and biotin) is introduced to the modified lysine via click chemistry. (**B**) Structures of NAS-K1 and NAS-K2 used in the previous study (*21*). The IC_{50} values are shown in the parentheses.

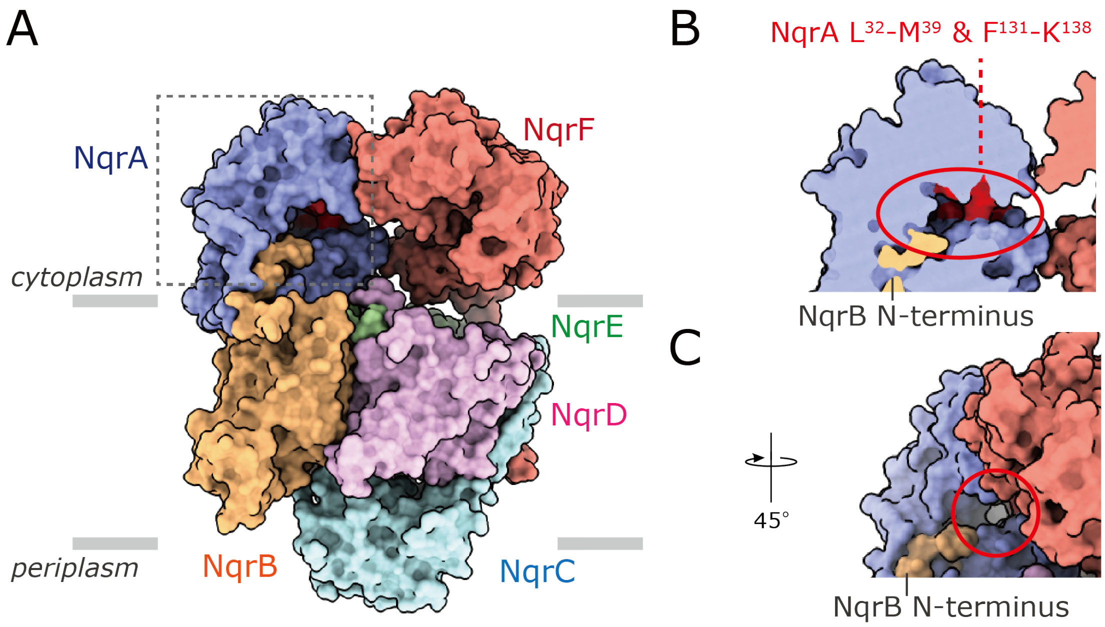

Supplementary Fig. 10: The binding site of the UQ head-ring in the cryo-EM structure. (**A**) The binding site of the UQ head-ring in NqrA is indicated in *red* in the current cryo-EM structure of Na^+-NQR without bound inhibitor. (**B**) The close-up view of the site (marked by a *red circle*). (**C**) The view of the site from a different angle.

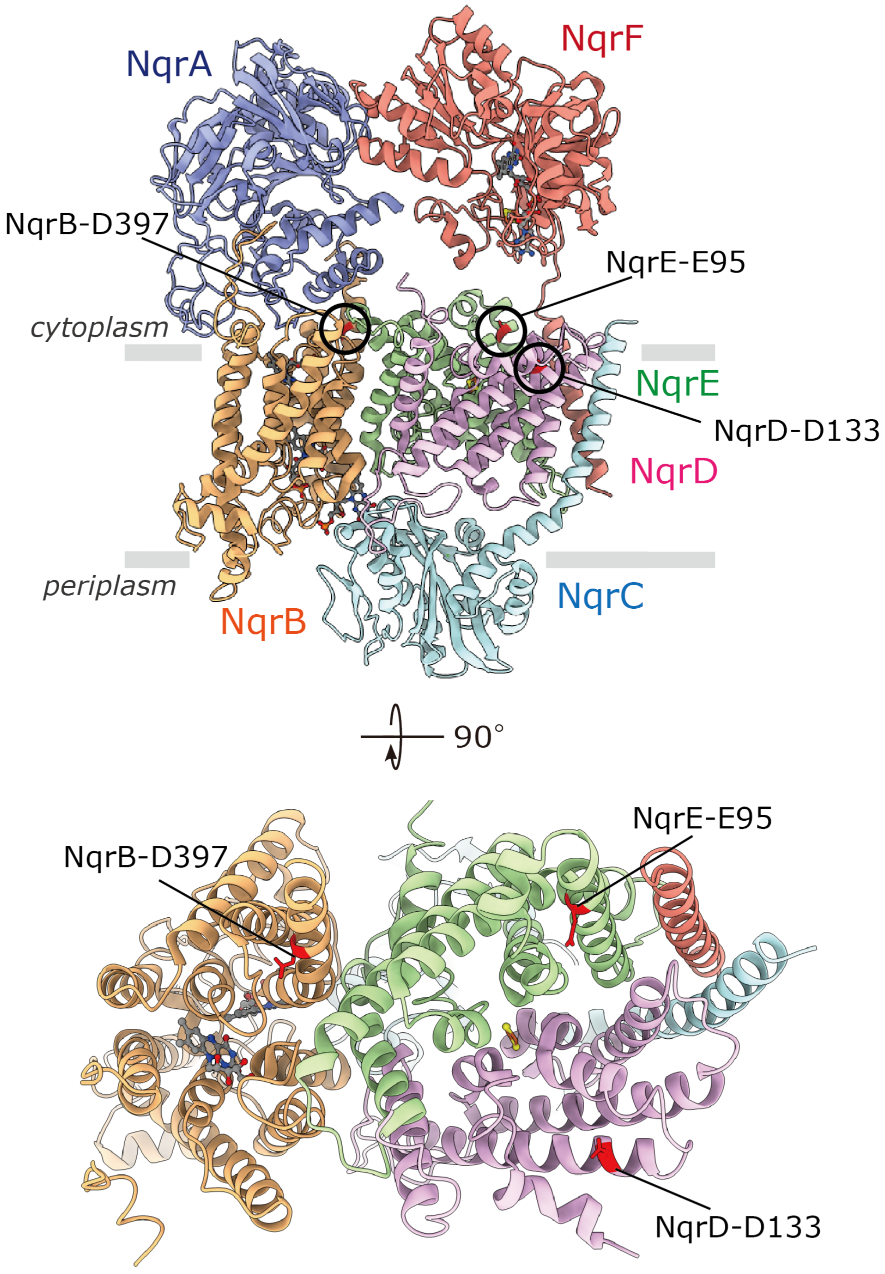

Supplementary Fig. 11: Locations of NqrB-Asp397, NqrD-Asp133, and NqrE-Glu95 in the cryo-EM structure of Na^+-NQR. These residues, which are important for Na^+ uptake, are highlighted in the figure. For clarity, the hydrophilic domains of NqrA, F, and C subunits are not shown in the lower panel.

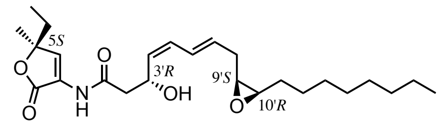

korormicin A (IC_{50} = 5.0 nM)

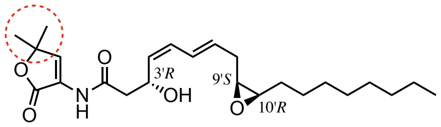

compound D1 (IC_{50} = 9.4 nM)

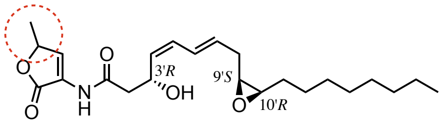

compound D2 (IC_{50} = 92 nM)

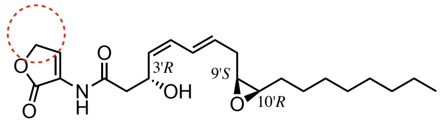

compund D3 (IC_{50} = 2700 nM)

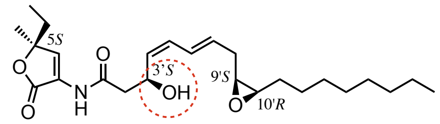

compound D4 (IC_{50} = 270 nM)

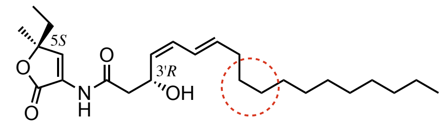

compound D5 (IC_{50} = 23 nM)

Supplementary Fig. 12: Structure-inhibition relationship of korormicin A derivatives. Structure-inhibition relationship of korormicin A derivatives (*19*). The IC_{50} value is the molar concentration needed to reduce the NADH-UQ_1 oxidoreductase activity of the isolated Na^+-NQR (1.0 nM) by 50%.

Supplementary Table 1. Statistics for Cryo-EM data, refinement, and validation of Na^+-NQR.

	Na^+-NQR		
**Data collection and processing**			
Magnification		81,000	
Voltage (kV)		300	
Total dose ($e^-/Å^2$)		65	
Defocus range (μm)		-0.8 to -2.0	
Pixel size (Å)		0.88	
Symmetry imposed		C1	
# of movies		9,027	
# of Initial particles		2,570,565	
**States**	**1**	**2**	**3**
EMDB ID	33242	33243	33244
PDB ID	7XK3	7XK4	7XK5
# of Final particle	82,790	80,882	72,234
Resolution (Å) FSC =0.143	3.1	3.1	3.1
**Model statistics**			
Model resolution (Å) FSC =0.5	3.1	3.1	
Model composition			
Non-hydrogen atoms	14,847	14,835	14,835
Residues	1,894	1,894	1,894
Ligands	FMN: 2, FES: 2, RBF: 1. FAD: 1, PEE: 1, CA: 1, LMT: 2,	FMN: 2, FES: 2, RBF: 1. FAD: 1, PEE: 2, CA: 1, LMT: 2,	FMN: 2, FES: 2, RBF: 1. FAD: 1, PEE: 1, CA: 1, LMT: 2,
Waters	0	0	0
Bond length (Å)	0.006	0.006	0.005
Bond angles (°)	0.800	0.846	0.787
Clash score	6.16	5.77	5.60
MolProbity score	1.56	1.43	1.51
EMRinger score	3.79	2.68	2.78
Rotamer outliers (%)	0.35	0.47	0.45
Ramachandran plot			
Favored (%)	96.55	97.40	96.76
Allowed (%)	3.29	2.50	3.13
Outlier (%)	0.16	0.11	0.11

Supplementary Table 2. Statistics for Cryo-EM data, refinement, and validation of Na^{+}-NQR^{AD42} and Na^{+}-NQR^{KA}.

	Na^{+}-NQR^{AD42}	Na^{+}-NQR^{KA}
**Data collection and processing**		
Magnification	81,000	
Voltage (kV)	300	
Total dose (e^{-}/Å^{2})	60	
Defocus range (μm)	-0.8 to -2.0	
Pixel size (Å)	0.88	
Symmetry imposed	C1	
# of movies	11,430	2,814
# of Initial particles	2,944,013	721,914
EMDB ID	33245	33246
PDB ID	7XK6	7XK7
# of Final particle	54,284	50,444
Resolution (Å) FSC =0.143	3.0	2.9
**Model statistics**		
Model resolution (Å) FSC =0.5	3.0	3.0
Model composition		
Non-hydrogen atoms	15,124	15,187
Residues	1,919	1,921
Ligands	AUD:1, FMN: 2, FES: 2, RBF: 1. FAD: 1, PEE: 2, CA: 1, LMT: 2,	KRR: 1, FMN: 2, FES: 2, RBF: 1. FAD: 1, PEE: 2, CA: 1, LMT: 2,
Waters	49	35
Bond length (Å)	0.006	0.012
Bond angles (°)	0.850	1.227
Clash score	4.69	4.96
MolProbity score	1.48	1.36
EMRinger score	3.68	4.09
Rotamer outliers (%)	0.58	0.57
Ramachandran plot		
Favored (%)	96.37	97.48
Allowed (%)	3.42	2.36
Outlier (%)	0.21	0.05

Supplementary Table 3. Comparison of the r.m.s.d values for each subunit between the cryo-EM-structure (this study) and crystallographic structure (*16*). The values were calculated using UCSF chimera. The distance between each Cα has been pruned by 5 Å.

**Subunit**	**r.m.s.d. (Å)**
**NqrA**	0.913
**NqrB**	1.349
**NqrC**	1.125
**NqrD**	2.107
**NqrE**	1.843
**NqrF**	1.317

Supplementary Discussion

Equilibrium binding model of ^{125}I-incorporated inhibitor and competitor

In the previous photoaffinity labeling study using photoreactive aurachin D-type inhibitors ([^{125}I]PAD-1 and [^{125}I]PAD-2, Supplementary Fig. 1B) with the isolated *V. cholerae* Na^+-NQR (*18*), we observed a unique competition behavior; that is, the labeling by these [^{125}I]-incorporated inhibitors rather than being competitively suppressed in the presence of excess other inhibitors (including their non-radioactive analogs PAD-1 and PAD-2), was *enhanced* when a molar ratio of ^{125}I-incorporated inhibitor to the enzyme is relatively low (<10). This unusual competitive behavior is difficult to reconcile with a simple scenario in which different inhibitors share a common binding site. To explain this, we proposed an equilibrium model for the binding of ^{125}I-incorporated inhibitor and competitor based on the assumption that there are two distinct inhibitor-bound states (i.e. one-inhibitor- and two-inhibitor-bound states), in which the yields of the labeling reaction are considerably different (ref. *18* for details). While we cannot exclude other scenarios that would explain the unusual competitive behavior, this model accounted for the consecutive changes in the nature of the competition (from enhancement to suppression) as the concentration of competitor increases. However, because the present cryo-EM structures provided no evidence for the existence of two distinct binding sites for inhibitor, the equilibrium model must be corrected.

The present study revealed that, while the N-terminal region (Gly2−Leu26) of NqrB is disordered in the absence of inhibitor, the binding of inhibitor gives rise to a distinct conformation of this region (Fig. 6A and 6B). It is, therefore, likely that the bound inhibitor shapes the region into its own binding cavity reflecting its chemical framework (the so-called “induced-fit” binding). Given that the inhibitor binding is a reversible equilibrium process, the binding cavity of the inhibitor may exist in, at least, two different conformations in equilibrium: one, disordered and indefinite conformation with a low affinity to inhibitor and the other, distinct fixed conformation with a high affinity to inhibitor. The latter would correspond to the binding cavity identified by cryo-EM (Fig. 6A and 6B). Based on this idea (namely, two different conformations of a single binding cavity), here we produced a new equilibrium binding model to explain the unique competitive behavior between ^{125}I-incorporated inhibitor (**[^{125}I]I**) and competitor (**C**), as shown in Supplementary Fig. 13. Note that although there could be many consecutive intermediates between the two extreme conformations, to simplify the analysis we have assumed only two conformations.

Since the radioactivity incorporated into the enzyme by photolysis is proportional to the concentration of the enzyme-^{125}I-incorporated inhibitor complex (**F-[^{125}I]I** complex) regardless

of the labeling reaction yields, we estimated the concentrations of **F-[^{125}I]I** complex by this equilibrium model. The "**D**" and "**F**" forms stand for the *disordered* and *fixed* conformations,

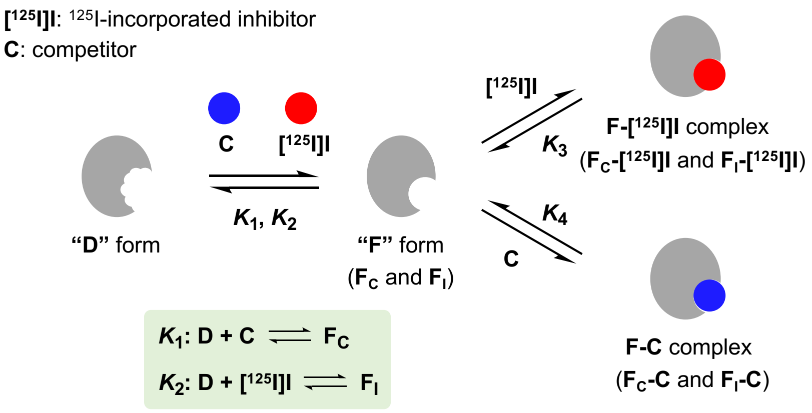

Supplementary Fig. 13. Schematic presentation of the equilibrium binding model based on two different conformations of a single binding cavity ("**D**" and "**F**" represent the disordered and fixed conformations, respectively).

respectively, of the binding cavity in the NqrB subunit. As the conformation of the cavity varies slightly depending on individual bound inhibitors, the **F** form takes two conformations in the strict sense: $\mathbf{F}_\mathrm{C}$ (competitor-bound conformation) and $\mathbf{F}_\mathrm{I}$ ([^{125}I]I-bound conformation). Therefore, regarding the equilibrium constants K_1 and K_2, we consider the following three cases (models 1–3). In the model 1, not only the conformations of $\mathbf{F}_\mathrm{I}$ and $\mathbf{F}_\mathrm{C}$ but also K_1 and K_2 are identical. This case corresponds to the competition between ^{125}I-incorporated inhibitor and its non-radioactive (cold) analog (*18*). In the model 2, the conformations of $\mathbf{F}_\mathrm{I}$ and $\mathbf{F}_\mathrm{C}$ are identical but K_1 and K_2 are different. In the model 3, the conformations of $\mathbf{F}_\mathrm{I}$ and $\mathbf{F}_\mathrm{C}$ and the equilibrium constants K_1 and K_2 are both different. This case corresponds to the competition between ^{125}I-incorporated inhibitor and a different type of inhibitor. Since the models 2 and 3 are extended cases of the model 1, we first numerically solved the equilibrium equations based on the model 1 ($K_1 = K_2$). The equilibrium compositions were calculated based on the kinetic simulations for a sufficiently long time by COMSOL Multiphysics® (COMSOL) under varying conditions. In the initial conditions of the simulation, the system contains only **D**, **[^{125}I]I**, and **C**.

For the current simulation, we set K_1, K_2, K_3, and K_4 to 100,000, 100,000, 1,000,000, and 1,000,000 mM^{-1} and the total concentration of **[^{125}I]I** to 10 nM under different total concentrations of both the enzyme (**D**) and competitor (**C**). Note that the total concentrations of **[^{125}I]I** (10 nM) and the enzyme (0.90, 9.0, 90, or 900 nM) were set to those used in the previous photoaffinity labeling experiments (*18*) throughout the following simulations. The employed equilibrium constants were estimated from the IC_{50} value for korormicin A (5.0 nM,

18), assuming that the affinity of **F** to the inhibitor (K_3 or K_4) is ten times larger than that of **D** (K_1 or K_2). Based on these assumptions, the changes of the concentration of individual species (**D**; enzyme in D-form, **F**; enzyme in F-form, **F-[^{125}I]I**; enzyme-^{125}I-incorporated inhibitor complex, **F-C**; enzyme-competitor complex) against the initial concentration of competitor ([**C**]$_0$) for four different enzyme concentrations were simulated, as shown in Supplementary Fig. 14.

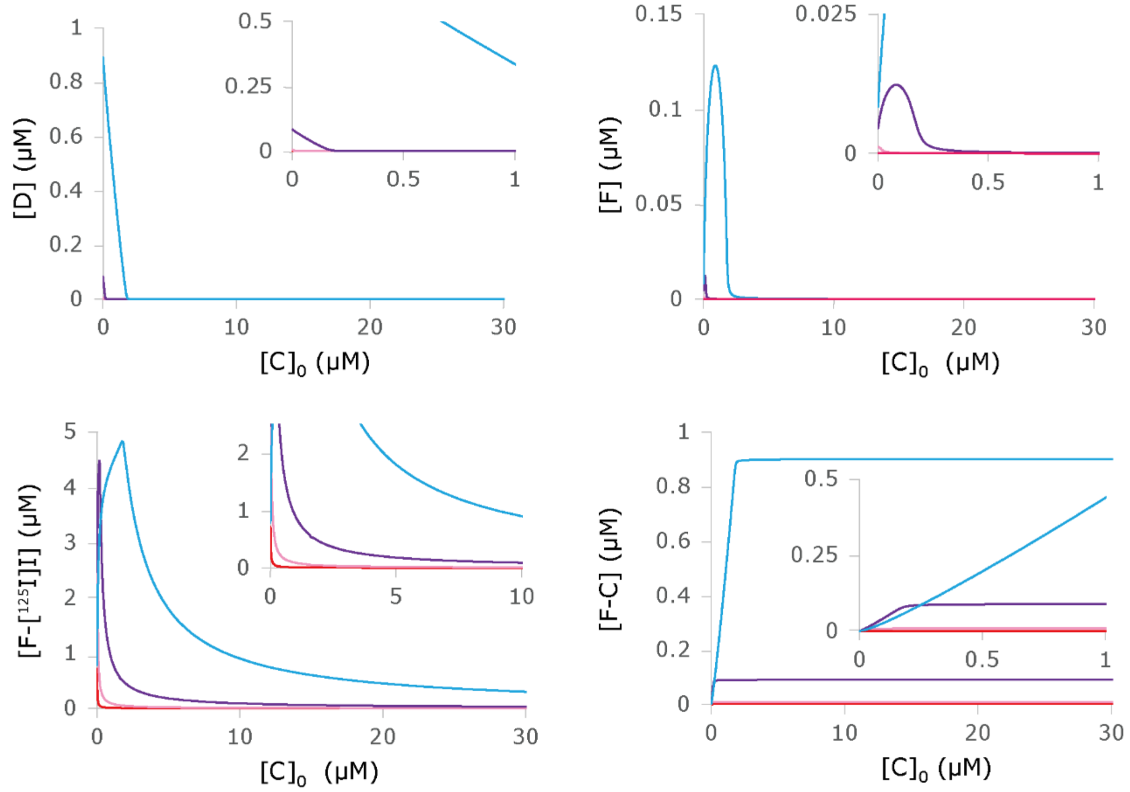

Supplementary Fig. 14. Changes of the concentration of individual species (enzyme in D-form (**D**), enzyme in F-form (**F**), enzyme-^{125}I-incorporated inhibitor complex (**F-[^{125}I]I**), enzyme-competitor complex (**F-C**)) against the initial concentration of competitor ([**C**]$_0$) with different enzyme concentrations. The concentration of Na^+-NQR: 0.9 (*red*), 9.0 (*pink*), 90 (*purple*), and 900 nM (*blue*).

Then, the concentrations of **F-[^{125}I]I** complex as a function of the concentrations of added competitor (**C**) were normalized by the concentration of **F-[^{125}I]I** when [**C**]$_0$ = 0, so that the value at zero added competitor is one (Supplementary Fig. 15); values below one indicate competitive suppression of the formation of **F-[^{125}I]I** complex, while values above one indicate enhancement. Our model can account for the consecutive changes of the effects of the competitor, from enhancement to suppression, as the concentration of the competitor increases in the case of a high concentration of the enzyme (900 nM). The enhancing effect became less clear with decrease in the enzyme concentrations. These simulation results are consistent with the experimental ones (*18*).

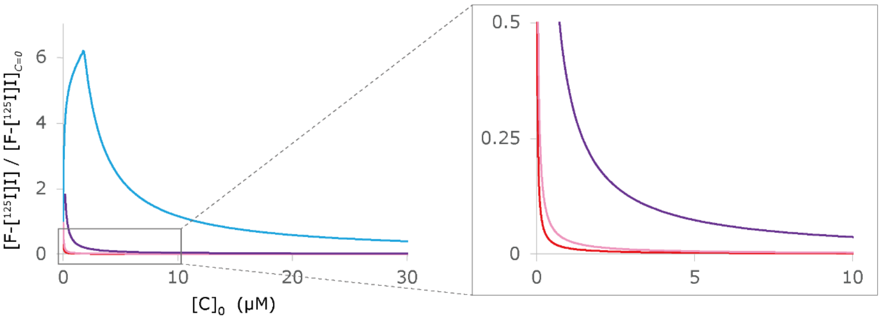

Supplementary Fig. 15. The concentration of **F-[^{125}I]I** complex as a function of the initial concentration of competitor (**C**), normalized so that the value at zero added competitor is one. The concentration of Na^+-NQR: 0.9 (*red*), 9.0 (*pink*), 90 (*purple*), and 900 nM (*blue*).

For reference, changes of the concentrations of **F-[^{125}I]I** complex when the parameters (K_1–K_4) are varied are shown in Supplementary Fig. 16. Similar tendencies in the changes of **F-[^{125}I]I** complex with those shown in Supplementary Fig. 15 were observed.

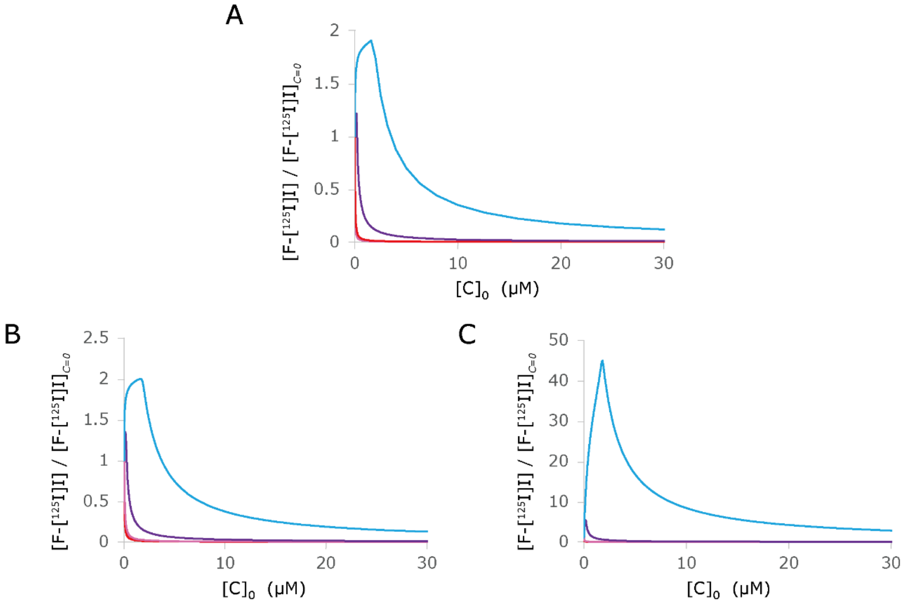

Supplementary Fig. 16. The concentration of **F-[^{125}I]I** complex as a function of the initial concentration of competitor (**C**). The simulations were conducted under three different conditions. Panel A: K_1, K_2, K_3 and K_4 were set to 100,000, 100,000, 10,000,000, and 10,000,000 mM^{-1}, respectively. Panel B: K_1, K_2, K_3 and K_4 were set to 10,000, 10,000, 1,000,000, and 1,000,000 mM^{-1}, respectively. Panel C: K_1, K_2, K_3 and K_4 were set to 1,000,000, 1,000,000, 1,000,000, and 1,000,000 mM^{-1}, respectively. The concentration of Na^+-NQR: 0.9 (*red*), 9.0 (*pink*), 90 (*purple*), and 900 nM (*blue*).

Next, we conducted the simulations according to model 2 ($K_1 \neq K_2$, but $\mathbf{F_C}$ is identical to $\mathbf{F_I}$). Based on the results of model 1, the K_1, K_2, K_3, and K_4 were set to 100,000, 200,000, 1,000,000, and 1,000,000 mM^{-1}, respectively. The consecutive changes of the effects of the competitor, from enhancement to suppression, were observed (Supplementary Fig. 17), as seen in the simulations based on model 1 (Supplementary Fig. 15).

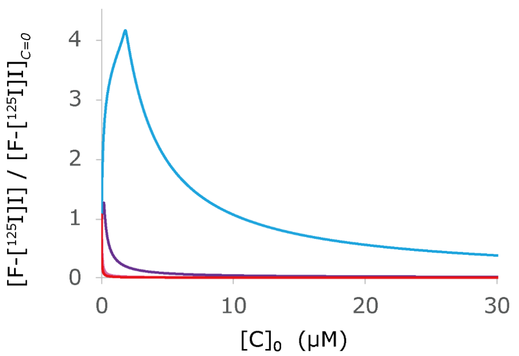

Supplementary Fig. 17. The concentration of **F-[^{125}I]I** complex as a function of the initial concentration of competitor (**C**), calculated based on the model 2. The K_1, K_2, K_3 and K_4 were set to 100,000, 200,000, 1,000,000, and 1,000,000 mM^{-1}, respectively. The concentration of Na^+-NQR: 0.9 (*red*), 9.0 (*pink*), 90 (*purple*), and 900 nM (*blue*).

Finally, we conducted simulations according to model 3 ($K_1 \neq K_2$, and $\mathbf{F_C}$ is not identical to $\mathbf{F_I}$). As the conformations of the $\mathbf{F_C}$ and $\mathbf{F_I}$ forms are different in this case, K_3 and K_4 increase twofold as follows:

$$K_{3\text{-}1}\text{: } F_C + [^{125}I]I \rightleftharpoons F_C\text{-}[^{125}I]I$$

$$K_{3\text{-}2}\text{: } F_I + [^{125}I]I \rightleftharpoons F_I\text{-}[^{125}I]I$$

$$K_{4\text{-}1}\text{: } F_C + C \rightleftharpoons F_C\text{-}C$$

$$K_{4\text{-}2}\text{: } F_I + C \rightleftharpoons F_I\text{-}C$$

We conducted the simulation by setting K_1, K_2, $K_{3\text{-}1}$, $K_{3\text{-}2}$, $K_{4\text{-}1}$, and $K_{4\text{-}2}$ to 100,000, 200,000, 1,000,000, 2,000,000, 1,000,000, and 2,000,000 mM^{-1}, respectively. Again, similar tendencies in the changes of the concentration of **F-[^{125}I]I** complex (a sum of $\mathbf{F_C}$**-[^{125}I]I** and $\mathbf{F_I}$**-[^{125}I]I**) with those simulated by model 1 were observed (Supplementary Fig. 18).

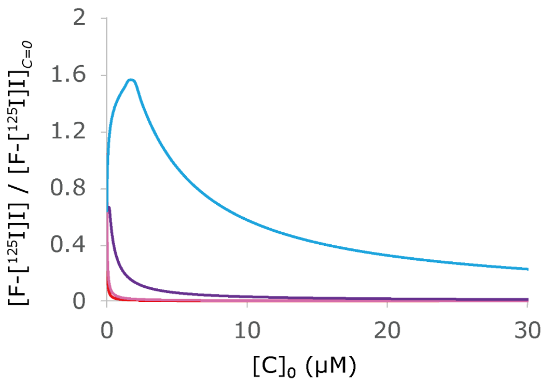

Supplementary Fig. 18. The concentration of **F-[^{125}I]I** complex as a function of the initial concentration of competitor (**C**), calculated based on the model 3. The K_1, K_2, $K_{3\text{-}1}$, $K_{3\text{-}2}$, $K_{4\text{-}1}$, and $K_{4\text{-}2}$ were set to 100,000, 200,000, 1,000,000, 2,000,000, 1,000,000, and 2,000,000 mM^{-1}, respectively. The concentration of Na^+-NQR: 0.9 (*red*), 9.0 (*pink*), 90 (*purple*), and 900 nM (*blue*).

In conclusion, the unusual competitive behavior observed in the previous photoaffinity labeling study (*18*) can be accounted for by the equilibrium model based on the idea of two different conformations of a single binding cavity. The extents of enhancing or suppressing effects vary depending on the parameters (K_1–K_4) that are determined by the individual chemical nature of ^{125}I-incorporated inhibitors and competitors used. Therefore, small differences in the effects among different pairs of ^{125}I-incorporated inhibitor and competitor, which were observed in the labeling experiments (*18*), would easily be accounted for. The current structural study led to this new equilibrium model, both by ruling out the presence of two distinct binding sites, and suggesting the possibility of multiple conformations of a single binding site with different affinities.

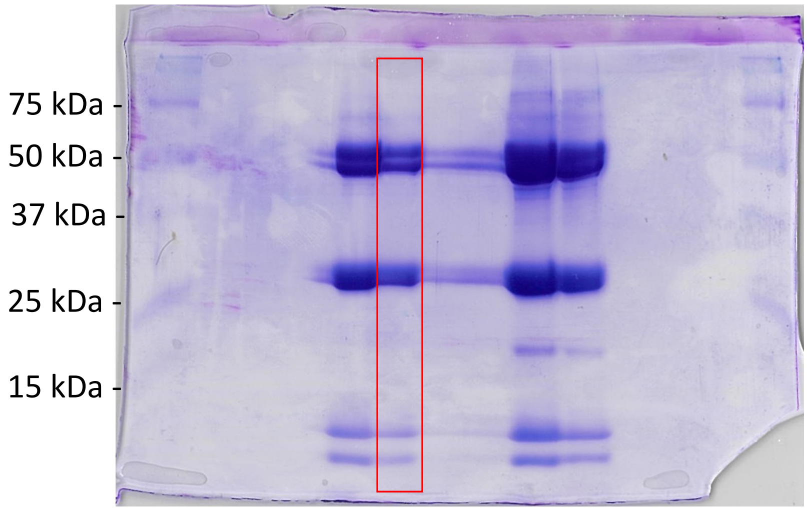

CBB stain

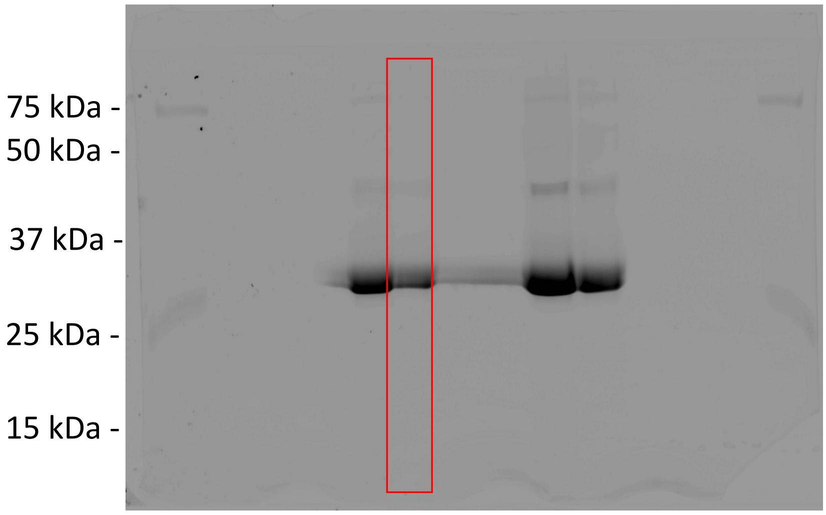

FMN

Uncropped gel scans of the purified Na^{+}-NQR (used in Supplementary Fig.4)
