# Supplementary material for: Cryo-EM structures of Na+-pumping NADH-ubiquinone oxidoreductase from Vibrio cholerae
Source: Nat Commun. 2022 Jul 26;13:4082. doi: 10.1038/s41467-022-31718-1 (PMC9325719; doi:10.1038/s41467-022-31718-1)
Supplement: Supplementary file 2 — Description of Additional Supplementary Files [file 41467_2022_31718_MOESM2_ESM.pdf]

File name: Supplementary Movie 1

Description: The flexibility of cytoplasmic domain of the NqrF subunit.
