# Supplementary material for: Cryo-EM structures of Na+-pumping NADH-ubiquinone oxidoreductase from Vibrio cholerae
Source: Nat Commun. 2022 Jul 26;13:4082. doi: 10.1038/s41467-022-31718-1 (PMC9325719; doi:10.1038/s41467-022-31718-1)
Supplement: Supplementary file 4 — Reporting Summary [file 41467_2022_31718_MOESM4_ESM.pdf]

nature portfolio

Corresponding author(s): Hideto Miyoshi

Last updated by author(s): June 15, 2022

Reporting Summary

Nature Portfolio wishes to improve the reproducibility of the work that we publish. This form provides structure for consistency and transparency in reporting. For further information on Nature Portfolio policies, see our Editorial Policies and the Editorial Policy Checklist.

Statistics

For all statistical analyses, confirm that the following items are present in the figure legend, table legend, main text, or Methods section.

n/a	Confirmed	
☒	☐	The exact sample size (*n*) for each experimental group/condition, given as a discrete number and unit of measurement
☐	☒	A statement on whether measurements were taken from distinct samples or whether the same sample was measured repeatedly
☒	☐	The statistical test(s) used AND whether they are one- or two-sided<br>*Only common tests should be described solely by name; describe more complex techniques in the Methods section.*
☒	☐	A description of all covariates tested
☒	☐	A description of any assumptions or corrections, such as tests of normality and adjustment for multiple comparisons
☒	☐	A full description of the statistical parameters including central tendency (e.g. means) or other basic estimates (e.g. regression coefficient) AND variation (e.g. standard deviation) or associated estimates of uncertainty (e.g. confidence intervals)
☒	☐	For null hypothesis testing, the test statistic (e.g. *F*, *t*, *r*) with confidence intervals, effect sizes, degrees of freedom and *P* value noted<br>*Give P values as exact values whenever suitable.*
☒	☐	For Bayesian analysis, information on the choice of priors and Markov chain Monte Carlo settings
☒	☐	For hierarchical and complex designs, identification of the appropriate level for tests and full reporting of outcomes
☒	☐	Estimates of effect sizes (e.g. Cohen's *d*, Pearson's *r*), indicating how they were calculated

Our web collection on statistics for biologists contains articles on many of the points above.

Software and code

Policy information about availability of computer code

Data collection	SerialEM v3.8.5
Data analysis	Cryosparc v3.2.0 or 3.3.1, topaz v0.2.3, Coot v0.9.2, Phenix v1.16, UCSF chimeraX

For manuscripts utilizing custom algorithms or software that are central to the research but not yet described in published literature, software must be made available to editors and reviewers. We strongly encourage code deposition in a community repository (e.g. GitHub). See the Nature Portfolio guidelines for submitting code & software for further information.

Data

Policy information about availability of data

All manuscripts must include a data availability statement. This statement should provide the following information, where applicable:

- Accession codes, unique identifiers, or web links for publicly available datasets
- A description of any restrictions on data availability
- For clinical datasets or third party data, please ensure that the statement adheres to our policy

"The data that supports the study's findings" are available in a publicly accessible repository. The cryo-EM maps have been deposited in the EMDB under accession codes, 33242, 33243, 33244, 33245, and 33246. The consensus maps for Na+-NQR, Na+-NQRAD42, and Na+-NQRKA were also deposited as additional maps in the depositions, 33242, 33245, and 33246. The atomic models have been deposited in the Protein Data Bank under accession codes, 7XK3, 7XK4, 7XK5, 7XK6, and 7XK7.

The initial model for model building is accessible in PDB under accession number 4P6V. The data that support the findings of this study are available from the corresponding author upon reasonable request.

Human research participants

Policy information about studies involving human research participants and Sex and Gender in Research.

Reporting on sex and gender	This study does not contain any analysis where human research participants are needed.<br>We did not use the terms "sex" and "gender" in the manuscript.
Population characteristics	This study does not contain any analysis where human research participants are needed.
Recruitment	This study does not contain any analysis where human research participants are needed.
Ethics oversight	This study does not contain any analysis where human research participants are needed.

Note that full information on the approval of the study protocol must also be provided in the manuscript.

Field-specific reporting

Please select the one below that is the best fit for your research. If you are not sure, read the appropriate sections before making your selection.

☒ Life sciences ☐ Behavioural & social sciences ☐ Ecological, evolutionary & environmental sciences

For a reference copy of the document with all sections, see nature.com/documents/nr-reporting-summary-flat.pdf

Life sciences study design

All studies must disclose on these points even when the disclosure is negative.

Sample size	The sample sizes of the cryo-EM datasets were judged by the resolution of the obtained maps. The resolution was estimated from independent 3D reconstruction. Because the resolutions were high enough (around 3 angstrom), we judged the sample size is enough.<br>The numbers of movies and particles used for this study are summarized in the Supplementary Figs. 1 and 2 and Supplementary Tables 1 and 2.
Data exclusions	Particles belong to bad classes in 2D and 3D classification were excluded.
Replication	In the structural analysis, we obtained almost the same structure from different three conditions (without inhibitor and with korormicin A or aurachin D-42 bound to the NqrB subunit), indicating the good reproducibility. In addition, the selected particle images were separated into halves and used for final 3D reconstruction respectively. The obtained two half maps were used to estimate the overall resolution. The estimated resolutions were high enough (around 3 angstrom). These results indicate the good reproducibility of the datasets.<br>In Supplementary Fig. 4, gel images are representative of three different enzyme preparations. UV trace is representative of two separate assays for each of three different enzyme preparations. All replications were successful.
Randomization	This is not relevant to structural determination using cryoEM, because no grouping is needed.
Blinding	Investigators were not blinded to the data, but the three datasets were analyzed independently. Because the obtained density maps from them were similar, we judged that there are no bias on our results.

Reporting for specific materials, systems and methods

We require information from authors about some types of materials, experimental systems and methods used in many studies. Here, indicate whether each material, system or method listed is relevant to your study. If you are not sure if a list item applies to your research, read the appropriate section before selecting a response.

Materials & experimental systems

n/a	Involved in the study
☒	☐ Antibodies
☒	☐ Eukaryotic cell lines
☒	☐ Palaeontology and archaeology
☒	☐ Animals and other organisms
☒	☐ Clinical data
☒	☐ Dual use research of concern

Methods

n/a	Involved in the study
☒	☐ ChIP-seq
☒	☐ Flow cytometry
☒	☐ MRI-based neuroimaging
